# Exploitation of an ant-plant mutualism by a cavity-nesting wasp

**DOI:** 10.7717/peerj.20984

**Published:** 2026-04-15

**Authors:** Dan Lestina, Mickal Houadria, Jasmine A. Gavin, Arthur Y.C. Chung, Kalsum Mohd Yusah, Michal Rindos, Klára Schlosserová, Heike Feldhaar, Tom M. Fayle

**Affiliations:** 1 Faculty of Science, University of South Bohemia, Ceske Budejovice, Czech Republic; 2 Biology Centre, Czech Academy of Sciences, Ceske Budejovice, Czech Republic; 3 Nature Conservation Agency of the Czech Republic, Prague, Czech Republic; 4 Institute of Tropical Biology and Conservation, Universiti Malaysia Sabah, Kota Kinabalu, Sabah, Malaysia; 5 Forest Research Centre, Forestry Department, Sandakan, Sabah, Malaysia; 6 Jodrell Laboratory, Royal Botanic Gardens Kew, Richmond, United Kingdom; 7 Faculty of Forestry and Wood Sciences, Czech University of Life Sciences Prague, Prague, Czech Republic; 8 Second Faculty of Medicine, Prague, Czech Republic; 9 Bayreuth Center of Ecology and Environmental Research (BayCEER), University of Bayreuth, Bayreuth, Germany; 10 School of Biological and Behavioural Sciences, Queen Mary University of London, London, United Kingdom

**Keywords:** Mutualism, Exploitation, Habitat change, Tropical forest, Ant-plant, Macaranga, Crematogaster, Dasyproctus, Oil palm, Sabah

## Abstract

**Background:**

Normally, *Crematogaster* ant symbionts protect their *Macaranga* tree hosts from herbivorous insects, and in return are provided with nutrition in the form of food bodies, as well as nesting space inside the empty or easily hollowed-out stems. This system has been extensively studied as a model of ant-plant mutualistic interactions. Here, we report a novel finding of an exploiter species, the cavity-nesting wasp *Dasyproctus agilis* (F. Smith, 1858), which hollows out *Macaranga* stems, and uses the resulting space to provision its larvae with adult Diptera. We predicted that the *Dasyproctus* wasps, which are commonly found in highly disturbed habitats elsewhere, would be more common in *Macaranga* stems in an agricultural habitat (oil palm plantation), than in heavily logged forest. We also predict that because wasps nest in the same plant structures as the mutualistic ants, plants with greater numbers of wasps should have smaller ant colonies (although note that ants might also exclude wasps). Any such reduction in ant colony size could then reduce the ability of the mutualistic ants to protect plants from herbivorous insects.

**Methods:**

We dissected 213 *Macaranga pearsonii* trees of all ants and wasps across the Malaysian state of Sabah to explore the extent of the phenomenon. To test our predictions, we then carried out further plot-based sampling of *M. pearsonii* plants in heavily logged forest and oil palm plantations in Sabah, Malaysian Borneo, and recorded the frequency of wasp colonisation. Next, we conducted plant-based sampling in oil palm plantations to explore the relationship between wasp abundance and *Crematogaster* ant colony size, and potential consequences for herbivory rates on *M. pearsonii* plants.

**Results:**

We found that the wasps exploit the cavities of multiple *Macaranga* species in disturbed habitats across a wide geographical area. Wasp colonisation of *M. pearsonii* was more frequent in oil palm plantations than in heavily logged forests. We also found that plants colonised by larger numbers of wasps had smaller protective ant colonies, although causal direction remained unclear. There was no evidence for reduced ant protection of plants from herbivores in conjunction with wasp colonisation. Taken together, our results indicate that anthropogenic habitat change can result in colonisation by an exploitative species that potentially disrupts mutualistic interactions through pre-emption of the resources traded by mutualistic partners.

## Introduction

Mutualisms, in which partners trade benefits, are vulnerable to environmental changes (Harrower & Gilbert, 2018). In obligate mutualisms, loss of one partner can lead to co-extinction of the other (Chomicki et al., 2019; Aslan et al., 2013). Even in facultative mutualisms, changes in costs and benefits can trigger a cascade of negative effects on the populations of partner species (Van Der Heide et al., 2021). These mutualistic interactions are also vulnerable to exploitation, either where a previously mutualistic partner stops providing benefits, but still continues to acquire benefits from its partner, or if non-mutualistic species can co-opt those benefits (Bronstein, 2001). More broadly, it is now becoming apparent that mutualisms (and other interactions) are context dependent; the existence and outcome of interactions depend on abiotic environmental conditions and on the presence of other species (Hoeksema & Bruna, 2015). For example, protection of plants from herbivory by their mutualistic ant symbionts decreases with elevation, because lower temperatures reduce ant activity (Plowman et al., 2017). However, the impact of the increased frequency of non-mutualistic species in anthropogenically disturbed areas on benefits for mutualistic partners remains poorly explored.

The interaction between *Macaranga* trees and their *Crematogaster* ant inhabitants is a classic model system for studying mutualistic relationships (Feldhaar et al., 2003; Fiala et al., 1989; Heil & McKey, 2003; Itino et al., 2001). Some species of *Macaranga* provide living space in their stems and food bodies to their ant partners (Dixit & Guicking, 2024; Fiala & Maschwitz, 1991; Fiala & Maschwitz, 1992a; Fiala & Maschwitz, 1992b). In return, the *Crematogaster* ants provide protection from herbivores, and even trim encroaching vegetation (Fiala et al., 1989). Although the degree of protection is variable between *Crematogaster* species (Fiala, 1996), the impacts of non-mutualistic species on the interaction have not yet been explored.

Anthropogenic habitat change is rapidly altering ecosystems worldwide (Beca et al., 2017; Dornelas et al., 2014; Stork et al., 2017). Oil palm plantation expansion in particular is a major threat to biodiversity in South-East Asia (Koh & Wilcove, 2008), causing loss of many of the species previously present in the affected areas. Conversion to oil palm plantation also changes the micro- and mesoclimate as well as a range other biotic and abiotic conditions for those species able to persist in the plantations and the surrounding area (Luskin & Potts, 2011). However, the impact of this habitat change on *Crematogaster-Macaranga* interactions is only now becoming apparent. For example, plants in oil palm plantations experience more herbivory than those in heavily logged forest (Houadria et al., 2020). Indeed, the impacts of anthropogenic habitat change on ant-plant mutualisms in tropical forests in general remains very poorly understood (Fayle et al., 2017).

In this study we focus on the plant species *Macaranga pearsonii* Merr., which depends on inhabitation by one of two ant species: *C. linsenmairi* and *C. borneensis* (Feldhaar, Maschwitz & Fiala, 2016). *M. pearsonii* plants are found across a range of habitats, although they are particularly common and locally abundant in more disturbed areas (Slik, Keßler & Welzen, 2003), potentially making them an ecologically important early successional tree. The species has pith-filled stems, in contrast to some ant-inhabited *Macaranga* species where stems grow hollow, and these are hollowed out by founding queens, with living space later being expanded by ant workers (Fiala & Maschwitz, 1992a). As with other myrmecophytic *Macaranga* species, *M. pearsonii* present food for their ant partners in the form of food bodies on the stipules (*e.g.*, Houadria et al., 2023).

In this study, we explore the occurrence of a newly reported species from the stems of *Macaranga* trees: the wasp *Dasyproctus agilis* (F. Smith, 1858) (see Materials & Methods for further details on the species). Despite numerous studies on myrmecophytic *Macaranga*, it has, to our knowledge, never been reported that a cavity-nesting wasp would occupy the living space intended for the mutualistic ants. In addition to reporting this phenomenon, we aim to explore the effect of wasp colonisation on the *Crematogaster-Macaranga* mutualism. As in any ecological study, this system is also influenced by a large number of external biotic and abiotic factors. However, we predict that the mutualistic ant-plant system will be more vulnerable to colonisation by these wasps in more anthropogenically disturbed habitats, since the species is reported from agricultural habitats elsewhere (Srivastava, Bhatnagar & Sukhani, 1990). Furthermore, because the wasps utilise the pith-filled stems that the plants provide as domatia for mutualistic ants, we hypothesise that wasp colonisation might reduce ant colony size. If this is the case, it could result in reduced protection from herbivores, since smaller ant colonies have been found to patrol less in another *Macaranga* species (Heil et al., 2001). To test these hypotheses, we investigate how wasp occurrence varies between heavily logged rainforest and oil palm plantation. We also test whether wasp abundance is correlated with decreased ant colonisation, and whether this coincides with reduced ant protection from herbivory.

### Materials & Methods

#### Study area

**Figure 1:**
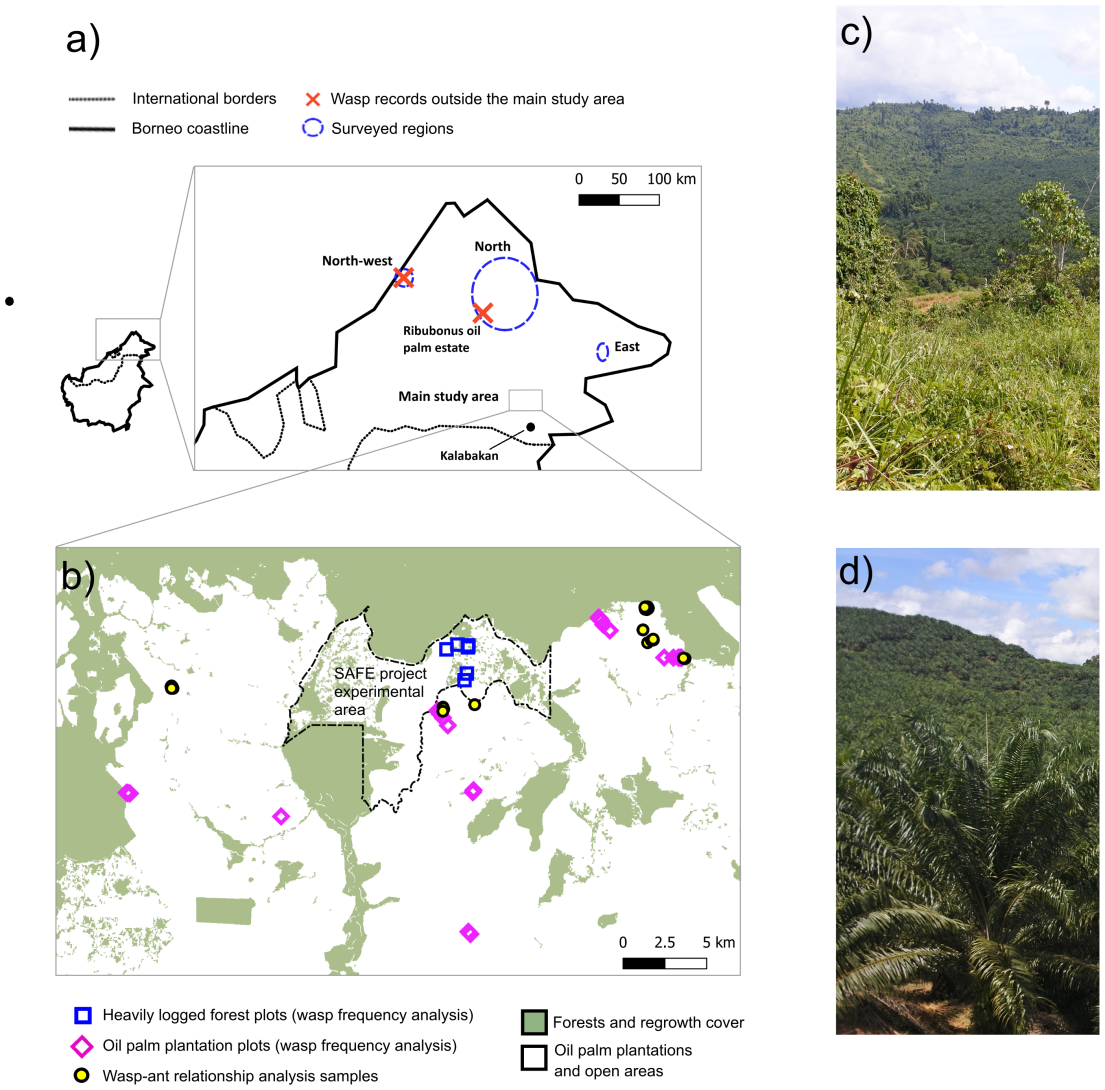
Field locations and illustrative photographs of the habitats surveyed for *Macaranga* plants. (A) Location of the main study area, other surveyed regions and of further reported wasp colonisation records. See Table S1 for details. (B) Detail of main study area, showing plots sampled to assess differences between habitats in wasp occupation, and locations of specimens sampled for relationships between wasp and ant colonisation of plants and the herbivory damage. Background forest cover data: ESA Global Forest Monitoring (Bourgoin et al., 2024). (C) Heavily logged forest in the study area. d) Oil palm plantation in the study area.

Field sampling of *M. pearsonii* plants was conducted between January 2017 and December 2018 in the state of Sabah, Malaysian Borneo (Figs. 1A, 1B). The region has a relatively a seasonal, tropical climate (mean monthly rainfall 185–285 mm, coldest monthly mean surface temperature 25.2 °C (January), warmest monthly mean surface temperature 26.4 °C (May); WBG Climate Change Knowledge Portal, 2021). The majority of forests in the state have undergone multiple rounds of logging (Fig. 1C), while the dominant agricultural land use is oil palm plantation monoculture (Reynolds et al., 2011; Fig. 1D). The main study area consists of the Stability of Altered Forest Ecosystems project area (SAFE; Ewers et al., 2011) and surrounding agricultural landscape (Fig. 1B). All site access and export of specimens was approved by the Sabah Biodiversity Council (License numbers: JKM/MBS.1000-2/2JLD.6(62), JKM/MBS.1000-2/2JLD.6(69), JKM/MBS.1000-2/2JLD.5(2), JKM/MBS.1000-2/2JLD.5(11), JKM/MBS.1000-2/2JLD.7(152), JKM/MBS.1000-2/3JLD(106)).

#### The cavity-nesting wasp *Dasyproctus agilis*

*D. agilis* widely distributed throughout South, East and Southeast Asia (Leclercq, 2015). Its biology has not yet been studied in detail. However, previous work indicates that different species in the genus *Dasyprocus* share a general biology (Binoy, Kumar & Santhosh, 2021; Bowden, 1964; Hamm & Richards, 1926). They nest in the stems of various plants, primarily monocotyledons (*e.g.*, Liliaceae, Amaryllidaceae, Iridaceae; but also eudicots: Rubiaceae). Initial stem excavation occurs while the stems are still green and pulpy. *Dasyproctus* wasps are found on plants throughout the year, with no difference between dry and wet seasons. A single plant is commonly shared between multiple females, although always with separate entrances and associated cells (Hamm & Richards, 1926). Furthermore, the same nesting sites can be used by several generations (Bowden, 1964). The wasp egg is sealed in a cell with an adult dipteran prey caught by the female, on which the larva later feeds. Development of the larva takes only around five to six days, with the pre-pupal period lasting for approximately 24 h. Larvae pupate with their heads towards the entrance hole. Pupal development takes 25 to 30 days (Bowden, 1964; Hamm & Richards, 1926). The flying period of adult females mirrors the activity of the dipteran hosts (*e.g.*, Brachycera–Hybotidae, Stratiomyidae, *etc*.), during the earlier hours of the day and at sunset (Binoy, Kumar & Santhosh, 2021; Bowden, 1964).

#### Difference in wasp colonisation frequency between habitats

To test the difference in wasp occupation between heavily logged forest (mostly salvage logged 2013–15) and oil palm plantations, we utilised plants dissected as a part of another study (Houadria et al., 2020; note differences in sample sizes from this study due to different sample inclusion criteria). The plants were collected from 28 standardised 25 m by 25 m plots in a structured sampling design in five different plantations (*n* = 43 plants) and in several parts of the logged forest (*n* = 41 plants) in the main study area (Fig. 1B; Materials and Methods and Electronic Supplementary Material 1 of Houadria et al. (2020)). Presence/absence of wasps was recorded for each plant in each plot. Because these sites were not selected based on wasp occupation, this provides an estimate of wasp colonisation frequency. The difference in occupation frequency between the habitats was tested using a Fisher exact test.

#### The relationship between wasp abundance and ant colony size

Because wasps were rare in our heavily logged forest samples, we focused further sampling on seven oil palm plantation areas (Fig. 1B), where we observed high densities of occupied and unoccupied plants by ants and wasps. We exhaustively collected all *Macaranga* plants present in these selected areas (36 ha in total). We recorded length, diameter and relative position of each branch/stem deemed habitable by mutualistic *Crematogaster* ants or *Dasyproctus* wasps. We defined habitable plants as those containing a pith-filled hollow, which was generally all branches over 3.5 mm in diameter at the base, hereafter referred to as “sections”, for each plant up to 400 cm in height found in the focal areas (*n* = 125). Mean section length was 74.7 cm (range: 13.2–326.0 cm). For each plant, total stem length as a measure of the total habitable living space for both ants and wasps was calculated by summing the length of all sections. Plants were cut down and dissected, and for each section, an estimated ant abundance category was recorded (0: 0 individuals; I: 1 to 10; II: 11 to 100; III: 101 to 1,000; IV: more than 1,000). These categories were designed to represent newly founded, developing, and mature colonies, which we assumed to have contrasting effectiveness in mediating plant protection from herbivores. Furthermore, use of abundance category estimation, rather than counting individual ants, allowed a greater sample size. The number of *Dasyproctus* wasp larvae and pupae was recorded and summed to give total wasp abundance. We then modelled the ant abundance category at two scales: (1) between trees, to test the effects of the parameters on total ant abundance in each tree, and (2) within trees, to explore differences in occupancy of individual stem sections (the branches and the main stem). The latter is relevant because previous work in the same area has demonstrated that *M. pearsonii* plants in oil palm plantations tend to have ant colonies that are concentrated in a smaller number of branches (Houadria et al., 2020). For the between-tree analysis, we used an Ordinal Logistic Regression (*polr* function in the *MASS* R package Agresti, 2012), with total stem length, main stem diameter and wasp abundance as predictors, and ant colony size category as the response variable. This method is appropriate for modelling response variables that are ordered categories. For the within-tree analysis, we only included sections from trees with >1 section (*i.e.,* trees having one or more habitable branches apart from the main stem). We used a Cumulative Link Mixed Model (*clmm* function in the *ordinal* R package), with section length, section type (main stem or side branch) and wasp abundance as predictors, and tree individual as a random predictor. This method is appropriate for modelling response variables that are ordered categories, while accounting additionally for random predictors. For the within-tree analysis, section diameter was not used as a predictor, as it was highly correlated with section type, and we wanted to avoid multicollinearity in the models. For both the between-tree and within-tree analyses, we used the *dredge* function in the *MuMIn* package to generate models with all combinations of main effects. We did not include interactions in these models. We ranked models by corrected AIC score (*AICc*), and discuss all models for which Δ*AICc* from the best model is less than 2 as also having “substantial support” (p70, Burnham & Anderson, 2002). Of those models, any that were within Δ*AICc* <2 of the best model, but that comprised a single additional predictor variable compared to the best model were not considered (p131, Burnham & Anderson, 2002).

#### Wasp-ant segregation

To complement the previous analysis, which assumes directional causation, a null model of co-occurrence was run to assess whether ants and wasps were significantly segregated between trees. Degree of overlap between the ant abundance category (see above, 0-IV, maximum per tree) and wasp abundance (larvae plus pupae, log-transformed to align with ant abundance categories), was calculated as follows. Both wasp number and ant colony size were standardised by dividing values by the maximum recorded for that variable for any single tree resulting in values between 0 and 1. The minimum of the standardised wasp abundance and ant colony size was then taken as the metric of overlap for a single tree, and a mean value calculated across all trees. To understand whether the observed degree of overlap differed from what would be expected at random, we generated 10,000 randomisations of wasp and ant occurrence. Larger plants were more likely to be colonised by both ants and wasps (see ‘Results’). To account for this, we calculated the proportions of plants occupied by wasps across six quantile bins according to the total habitable living space of each tree. We then randomised the wasp abundances, accounting for tree size using a two-step process. For each tree we first ran a Bernoulli process (R function *rbern*) with the probability of success being equal to the observed proportion of trees occupied by wasps in that size bin. If the outcome of the Bernoulli process was 0, then no wasps were present in that tree for that simulation. If the outcome was 1, then a wasp abundance was chosen at random (with replacement) from the observed wasp abundances within the corresponding plant size bin. We then calculated the mean overlap metric for each simulation. The number of simulations with a mean overlap metric equal to or less than the observed one was counted and was divided by the number of simulations to generate a *p*-value. Note that this is a one tailed test, since we predicted that wasps and ants would be segregated, not aggregated.

#### Plant leaf area loss

The five youngest fully-grown leaves on each section collected were assigned to one of four categories (I: missing or dead tissue 0–5% of the original leaf area; II: 5–25%; III: 25–50%; IV: 50–99%). This measured damage recently suffered by the plant, over the lifespan of these young leaves. Leaf damage for each plant was calculated as the mean of the midpoint values of these categories (2.5%, 15%, 37.5%, 75%) for each leaf position within a section (1st to 5th fully formed leaf), and then taking the mean of those positions (*n* = 114). We modelled leaf damage score at the between-tree scale as predicted by total stem length, total wasp abundance and ant colony size category using Beta regression (Cribari-Neto & Zeileis, 2010). Model selection was conducted as described above.

#### Further records of wasp colonisation of *Macaranga*

To document the wider prevalence of this interaction, further records of wasp colonisation from other *Macaranga* species in Sabah are presented. We recorded more than 600 *Macaranga* trees during other fieldwork (D. Lestina et al., 2017–2019, unpublished data) in addition to the data presented above, and several other specimens during coincidental observations. Out of these combined data, there were 213 trees of myrmecophytic species (*M. pearsonii, M. winkleri, M. hypoleuca* and other myrmecophytic species from sect. *Pachystemon*) of suitable size for wasp colonisation which were thoroughly inspected (0.4 to 4 m in height; see Table S1). Documented wasp colonisations are presented here.

### Results

#### Differences in wasp colonisation frequency between habitats

**Figure 2:**
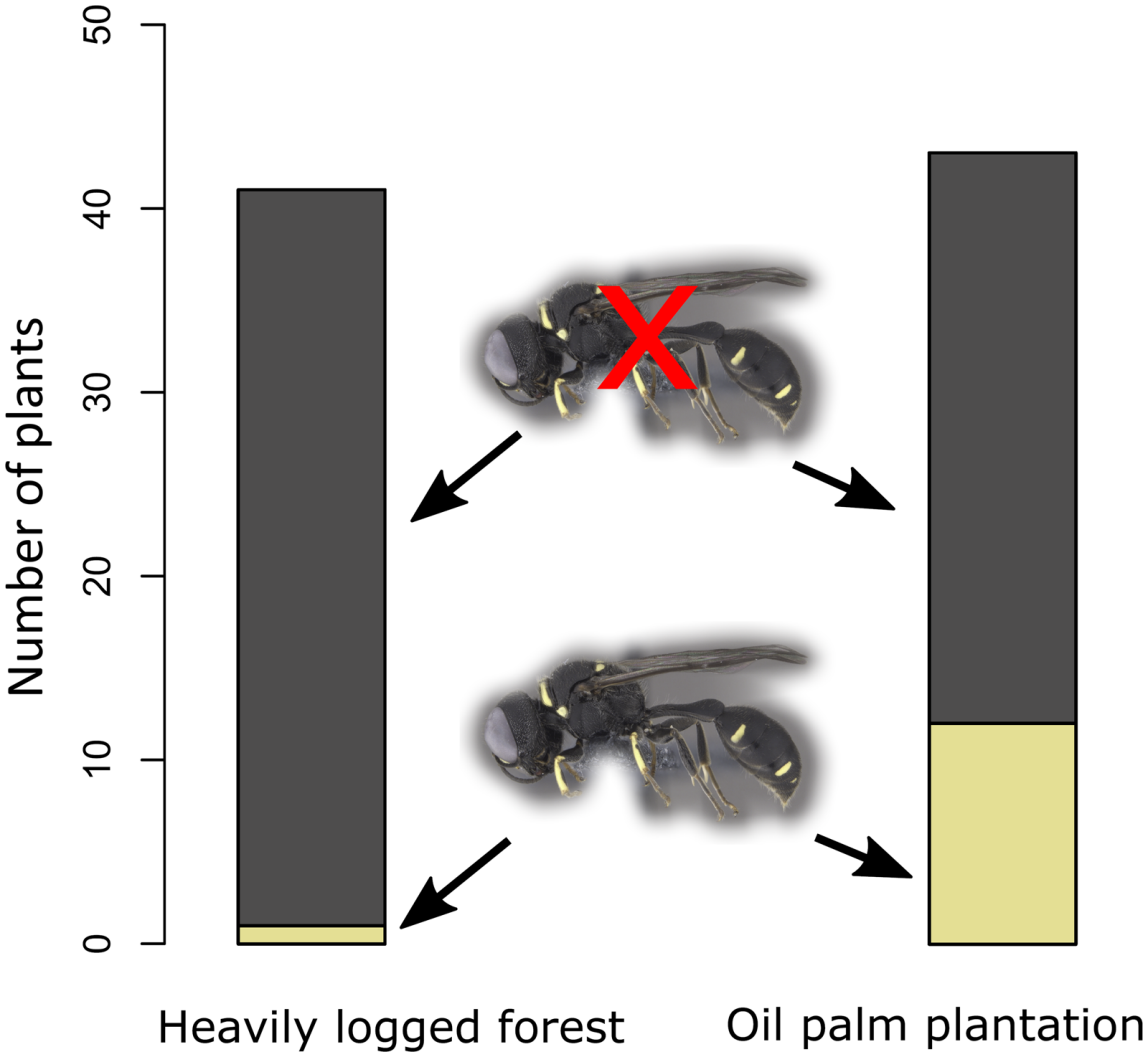
Wasp colonisation frequency in relation to habitat. Wasp colonisation frequency of *M. pearsonii* plants was greater in oil palm plantation than in heavily logged forest.

Wasp colonisation was significantly greater in oil palm plantations (12 of 43 trees) than in heavily logged forest (one of 41 trees; two-tailed Fisher exact test *p* = 0.002, Fig. 2).

#### The relationship between wasp abundance and ant colony size

**Figure 3:**
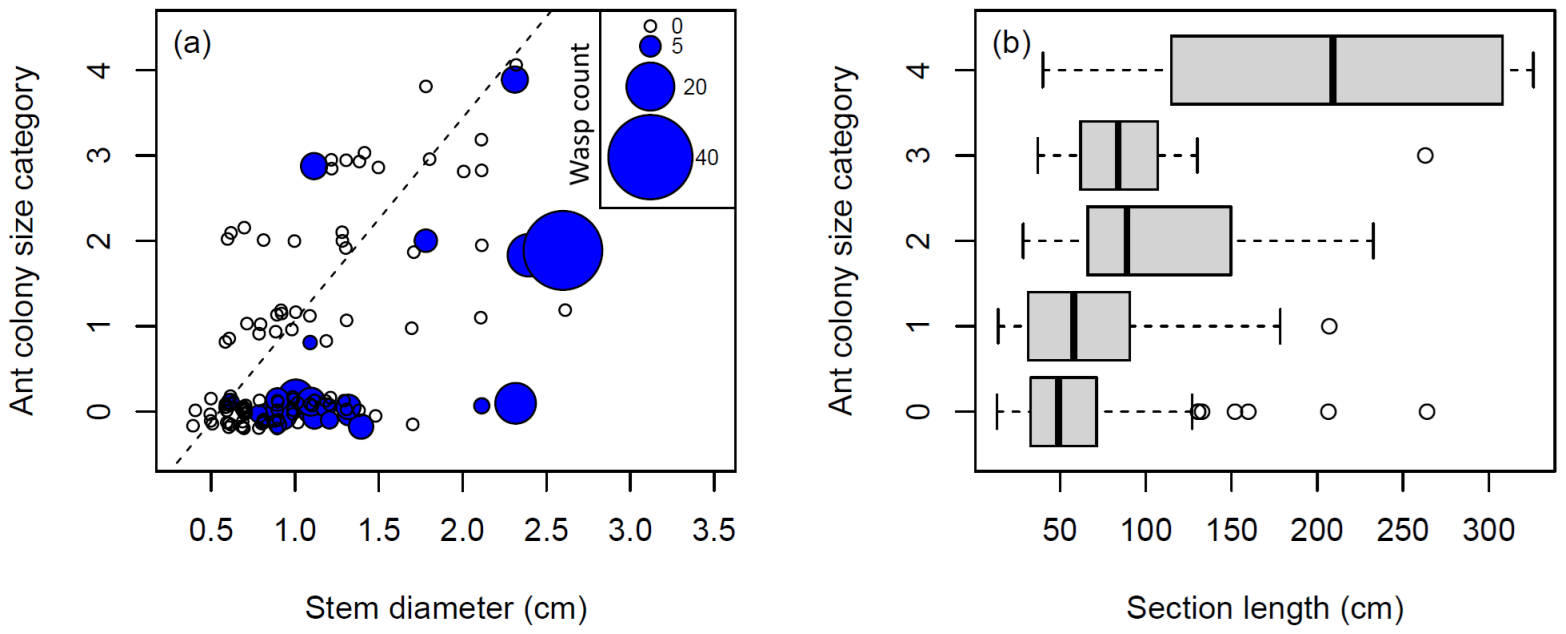
The relationship between *Macaranga* plant size, ant colony size, and wasp colonisation. (A) *M. pearsonii* plants colonised by wasps had smaller ant colonies when accounting for plant size in terms of stem diameter, when analysed at the between-plant scale. The broken line has been fitted by eye to illustrate the apparent limit of ant colony size when wasps are present *i.e.,* only one *M. pearsonii* plant with presence of wasps has an ant colony size above this line. Note that points have been jittered along both *x* and *y* axes in order to aid visualisation. (B) At the within plant scale, ant colony size was mainly predicted by the stem length, but not the abundance of wasps.

In oil palm plantations, at the between-plant scale, larger ant colonies were found in plants with greater branch/stem diameter and fewer wasps (Ordinal logistic regression: best model *AICc* = 248.6, *df* = 118,6; Table S2; Fig. 3A). An equally well-supported model (*AICc* = 248.9, Δ*AICc* = 1.3) also included total stem length as a predictor, but differed from the best model by only the addition of this single predictor, and was not considered further. The negative relationship between ant colony size and number of wasps was present regardless of the plant size predictors included in the model.

At the within-plant scale, the best model (Cumulative link mixed model: *AICc* = 319.4, *n* = 157; Table S3) indicated that more ants were found in longer sections (Fig. 3B). An equally well-supported model (*AICc* = 319.7, Δ*AICc* = 0.29) also included the binary predictor stem type, although it differed from the best model by only the addition of this single predictor, and so was not considered further. Wasp abundance was not included in either of the two best models (Δ*AICc* < 2).

#### Wasp-ant segregation

**Figure 4:**
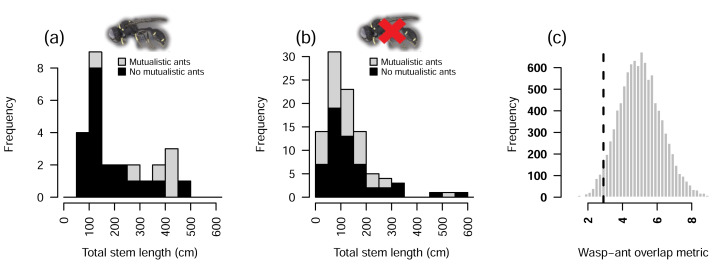
Null model analyses of co-occurrence between *Crematogaster* ants and *Dasyproctus* wasps in *Macaranga* plants. (A) When wasps were present, smaller plants in terms of total stem length were mostly uninhabited by mutualistic ants. (B) When wasps were absent, smaller plants were more often inhabited by mutualistic ants. Note that two plants with total stem lengths >600 cm are not plotted. (C) A null model shows less co-occurrence (vertical dashed line) between ants and wasps than would be expected at random (histogram, *n*_*sims*_ = 10, 000).

Observed overlap was significantly smaller than that of the null model (*p* = 0.035), indicating wasp-ant segregation (Fig. 4).

#### Plant leaf area loss

The best model for predicting leaf area loss included no predictors and only an intercept (Beta regression: *AICc* = −112.4, *n* = 113; Table S4). However, four other models were equally well-supported (Δ*AICc* < 2). Of these, three included only a single additional predictor (*i.e.,* a single predictor in total), and hence were not considered. The other included a positive relationship between wasp abundance and amount of herbivory (Fig. S1a), and a negative relationship between ant colony size and amount of herbivory (Fig. S1b).

#### Further records of wasp colonisation of *Macaranga*

Colonisation of *M. pearsonii* by a wasp, likely *Dasyproctus agilis* based on the general appearance of the nests and adults, was observed across our main study area near Kalabakan in southern Sabah and once in *M.* cf. *glandibracteolata* close to the NW coast of Sabah. We confirmed *D. agilis* colonisation at the Labuk river at the Ribubonus oil palm estate in northern (Fig. 1A, Table S1). We also found wasp nests in eight *M. hypoleuca* trees and two *M.* cf. *indistincta* trees and recorded another unidentified wasp species in *M. pearsonii* plants within the main study area. In other surveys, we found no wasps in the small numbers of myrmecophytic *Macaranga* plants of suitable size in and around forest fragments within oil palm plantations in northern and eastern Sabah (Fig. 1A, Table S1).

### Discussion

We observed wasp colonisation of *Macaranga* at widespread locations across Sabah and in multiple *Macaranga* species. *D. agilis* has previously been reported throughout SE Asia, and elsewhere in Borneo, although not in Sabah, and not specifically in *Macaranga* in any region (Leclercq, 2015). To our knowledge, this is also the first report of *D. agilis* using any member of the plant family Euphorbiaceae as a nesting site. Our results indicate either novel recent wasp colonisation of *Macaranga*, or lack of reporting of the phenomenon in numerous previous studies. The level of habitat degradation is not clear from most of the previous *Macaranga-Crematogaster* interaction studies, but we assume that these studies may have omitted disturbed habitats. This is despite the fact that such areas have become very widespread in the region in recent decades (Reynolds et al., 2011), and therefore might be more representative of *Macaranga* ecology today.

We demonstrate an increased colonisation of ant-plants by *D. agilis* in the more degraded habitat. Wasp colonisation was lower in heavily logged forest than in oil palm plantations. It is likely that these logged forests are relatively similar in terms of microclimate (Hardwick et al., 2015) and biota (Edwards et al., 2011) to primary forests, and hence may not be viable for colonisation by the wasps, particularly if *D. agilis* is adapted to more open habitats. Oil palm plantations, in contrast, show much greater biotic (Rembold et al., 2017) and abiotic (Luskin & Potts, 2011) homogeneity, with use of chemical fertilisers, herbicides and pesticides (Ashton-Butt et al., 2018). This may favour a widespread generalist species such as the wasp *D. agilis* (which has been reported in an agricultural crop elsewhere: Srivastava, Bhatnagar & Sukhani, 1990), rather than the co-evolved ant partner. This is because there is a greater likelihood that a widespread generalist will be pre-adapted to the novel environment presented by these highly modified habitats. The abundance of Diptera (the main prey for the wasp) in canopy and leaf litter is lower in oil palm than in logged forests (Turner, 2005). Hence availability of the wasp’s prey is unlikely to be driving the difference between habitats, unless the wasps are specialised on a prey taxon specific to oil palm plantations. It has been shown in another *Macaranga-Crematogaster* system that habitat change can affect which ant species occupies the tree, which could in turn influence resistance to wasp colonisation (Murase et al., 2003). However, previous results from our study system (Houadria et al., 2020) show that there is no difference between these habitats in the number of trees occupied by non-mutualistic ants or by either of the two mutualistic ant species. Therefore, we assume that the differences in wasp colonisation are not due to low ant occupancy caused by a lack of colonising ant queens of either species in the oil palm plantations. These previous results (Houadria et al., 2020) do show that for a given ant colony size, there is lower branch occupancy in oil palm plantations, which may be related to wasp occupancy of some of the branches. This could either be caused by another factor and the wasps could utilise this opportunity, or the lower branch occupancy could be caused by the wasp nests occupying some of the branches. The oil palm plantations that we sampled were run by large-scale commercial companies using standard practices. Hence these wasps may be potentially widespread, throughout Borneo and more broadly SE Asia, where large areas of plantation are managed in the same way.

**Figure 5:**
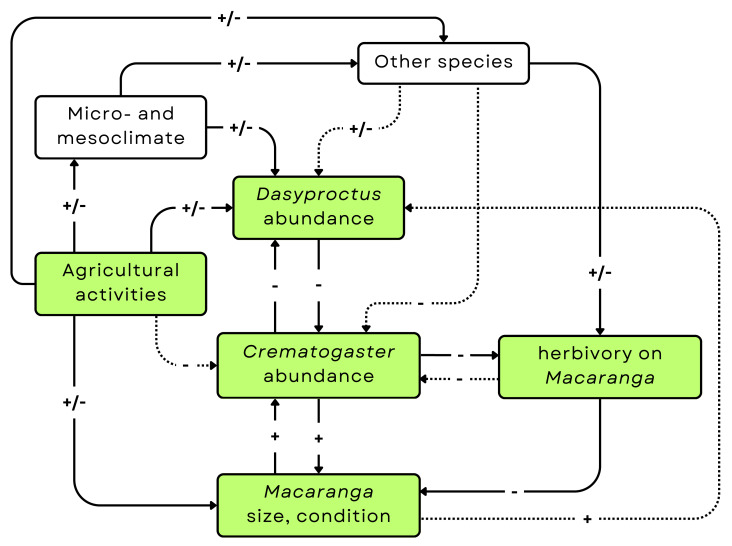
A summary of the current state of knowledge of the system, including our main findings. Green boxes indicate variables directly studied in some parts of our observations. Arrows indicate known relationships or relationships newly reported here (*i.e., Crematogaster-Dasyproctus*). Dashed lines indicate weaker or less certain influence.

We further explored the overlap of *D. agilis* nests with *Crematogaster* colonies and the possible influence of the exploitation of the domatia on herbivory damage on *M. pearsonii* (Fig. 5). Wasps and ants were segregated between individual plants, with the same result found regardless of whether we assumed causal direction (*i.e.,* using ordinal logistic regression or null modelling of co-occurrence). If wasp colonisation reduces the living space available for ants, this represents an instance of exploitation of a mutualism by a non-partner species, something previously observed in a range of systems (Bronstein, 2001). Indeed, degree of protection varies even between ant partners in other ant-plant systems, with some ant inhabitants apparently failing to protect plants at all (Edwards et al., 2010). Where plants have long-term evolutionary history with potentially cheating partners, they have often evolved adaptations to coerce partners into cooperating, such as the application of retaliatory sanctions (Edwards et al., 2006). However, if the *M. pearsonii* –*D. agilis* interaction is a novel one, then it is unlikely that the plants will have had time to evolve such adaptations. We assume that *Dasyproctus* wasps occupy fresh, not yet ant-colonised stems of *Macaranga*, in accordance with their behaviour in other plants (Bowden, 1964). However, previous work has demonstrated that even *Macaranga* plants with low ant activity (rather than completely unoccupied ones) are more likely to be inhabited by other insect species (Shimizu-kaya, Kishimoto-Yamada & Itioka, 2015), and hence these plants may be more susceptible to colonisation by wasps. This may also be the case for *Macaranga* plants in which ant colonies are concentrated in a smaller number of branches, as occurs in oil palm plantations (Houadria et al., 2020). Finally, this pattern might relate to some unmeasured biotic or abiotic driver, to which ants and wasps have opposing responses. For example, presence of natural enemies that target only one of the two groups, or differing thermal niches, combined with thermal heterogeneity of the habitat. Distinguishing these possibilities would require experimental manipulation of possible influencing factors, to complement the observational approach used in this exploratory study. The morphology of the entrance holes of ant- and wasp-inhabited stems that we observed suggests that ants generally do not recolonise *Macaranga* after *Dasyproctus* nesting. However, further study would be needed to address this point properly. If wasps do exclude ants from plants, then in the longer term we would expect the evolution of greater aggression from the ant partner towards this novel exploiter of the mutualism. This is despite the fact that the exploitation appears more benign than that observed in *Acacia* plants colonised by invasive ant species (Kamaru et al., 2024). We might also expect evolution of avoidance of ant-inhabited *Macaranga* plants by female wasps, if ant aggression is sufficient to reduce wasp reproductive success. The fact that the main prey of the wasp is Diptera, rather than a *Macaranga* natural enemy, suggests that plant partner switching from ants to wasps in response to anthropogenic change, as observed in other systems (Kiers et al., 2010), is unlikely here.

Unexpectedly, we found little evidence for a relationship between herbivory and either ant abundance or wasp abundance, although in both cases the weak relationship was in the expected direction. This is particularly surprising in terms of impacts of ant occupation, given previous reports of reduced herbivory in ant-occupied *Macaranga* generally (Fiala et al., 1994), and in *Macaranga* with larger ant colonies (Houadria et al., 2020). This also contrasts with evidence from a wider range of ant-plant systems showing less herbivory where ant abundance or species richness is higher (Fayle et al., 2012; Piovia-Scott, 2011; Soares et al., 2022). Our unexpected result could be partly explained by survivorship bias if heavily damaged plants rapidly die, and hence are not surveyed. However, a more likely explanation is that for the young plants we surveyed (mean height = 125 cm, 80% of plants <2 m in height), ant colonies were not yet large enough to influence herbivory and the effect could be stronger later in tree ontogeny, because adult *M. pearsonii* is considered dependent on its partner (Fiala et al., 1994). This also contrasts with patterns observed in a less specific mutualism between ants and epiphytic bird’s nest ferns (*Asplenium* spp.), where ant protection from herbivory does not vary, even between primary forest and oil palm plantation (Fayle et al., 2015). This is likely due to the lower specificity of the fern-ant interaction, with high levels of ant species turnover between habitats, relative to the highly specific *Crematogaster-Macaranga* interaction.

It is possible that use of pesticides influenced our results, since such impacts on mutualisms have been observed for plant–pollinator interactions (Kearns, Inouye & Waser, 1998). However, the plantation management reported no use of insecticides during our sampling periods. Use of herbicides would be clearly evident from the condition of the plants, as it was in several impacted areas elsewhere, which we did not consider for sampling. The negative correlation between wasp and ant abundances is unlikely to be driven by insecticides, since use of these would reduce abundances of both groups, resulting in positive correlations. Furthermore, we measured only leaf herbivory, but not stem damage caused by nesting wasps, which was sometimes substantial, and could lead to increased risk of stems breaking. Future work should examine the long-term impacts of wasp colonisation on the health and survival of *M. pearsonii* and its ant partners, and include experimental manipulations to determine causal direction.

This interaction may be significant in the broader oil palm ecosystem. If *D. agilis* is more likely to select highly abundant dipteran species, then this natural enemy has the potential to mediate intraspecific negative density dependence, and hence to reduce the abundances of those species. The potential for the wasps to control dipteran populations could also be explored, as this group can pose a public health risk, and can be found breeding at high abundances in empty fruit bunches in oil palm plantations (Fithri, 2021). However, if *D. agilis* becomes very widespread, it might have negative impacts on *Macaranga* populations, given the potential impacts of stem damage on plant health. Because *Macaranga* are common during the early stages of succession, and can be used during the initial stages of forest restoration (Changkija, Thakuria & Cynthia, 2023), spread of *D. agilis* could hinder forest regeneration.

## Conclusions

Here we present the first description of exploitation of the well-studied *Macaranga*-*Crematogaster* mutualism by the cavity-nesting wasp *Dasyproctus agilis*, which we found in several locations in disturbed habitats, suggesting vulnerability of the mutualistic system in these environments. We found that plants in the more disturbed habitat, oil palm plantation, had higher frequencies of wasp colonisation, which correlated with smaller ant colonies (Fig. 5). However, we did not find significant evidence of the effect of the wasp colonisation on the protection of the plants by the ants, as measured by the herbivory damage on the leaves. Taken together, our results indicate that anthropogenic disturbance has the potential to facilitate exploitation of existing mutualisms, with implications for ongoing co-evolutionary dynamics in novel ecosystems.

## Supplemental Information

10.7717/peerj.20984/supp-1 Supplemental Information 1 Numbers of myrmecophytic Macaranga trees of suitable size inspected in addition to the datasets used for testing of the hypotheses, and number of trees with recorded wasp nests All nests apart from one in *M. pearsonii* in the main study area were not distinguishable from *Dasyproctus agilis*, even though properly developed adults necessary for species identification were collected only from several trees in the main study area and from the specimen of M. pearsonii at the Labuk river in North Sabah. See Fig. 1 for a map of the regions and wasp records.

10.7717/peerj.20984/supp-2 Supplemental Information 2 Description of all models used in analyses of ant colony size and herbivory rate

10.7717/peerj.20984/supp-3 Supplemental Information 3 *Dasyproctus* wasp colonisation and *Crematogaster* colony size as predictors ofMacaranga herbivory damage Proportion of leaf area on *M. pearsonii* plants with herbivory damage as predicted by (a) number of wasps and (b) ant colony size category. Sample sizes for ant colony size categories: 0: n=69; 1: n=17; 2: n=13, 3: n=11, 4: n=3. Note that a model with no predictors and only an intercept had equally good support.

10.7717/peerj.20984/supp-4 Supplemental Information 4 Code and data for all analyses and plotting conducted

## Additional Information and Declarations

### Field Study Permissions

The following information was supplied relating to field study approvals (i.e., approving body and any reference numbers): Access to field work sites and export of biological specimens was approved by Sabah Biodiversity Council.

### Data Availability

The following information was supplied regarding data availability: All raw data and code for analysis and plotting of figures are available in the Supplemental Files.
