# Supplementary material for: Exploitation of an ant-plant mutualism by a cavity-nesting wasp
Source: PeerJ. 2026 Apr 15;14:e20984. doi: 10.7717/peerj.20984 (PMC13091584; doi:10.7717/peerj.20984)
Supplement: Supplemental Information 1 — All nests apart from one in M. pearsonii in the main study area were not distinguishable from Dasyproctus agilis, even though properly developed adults necessary for species identification were collected only from several trees in the main study area and from the specimen of M. pearsonii at the Labuk river in North Sabah. See Fig. 1 for a map of the regions and wasp records. [file peerj-14-20984-s001.pdf]

Supplemental Table S1. Numbers of myrmecophytic *Macaranga* trees of suitable size inspected in addition to the datasets used for testing of the hypotheses, and number of trees with recorded wasp nests. All nests apart from one in *M. pearsonii* in the main study area were not distinguishable from *Dasyproctus agilis*, even though properly developed adults necessary for species identification were collected only from several trees in the main study area and from the specimen of *M. pearsonii* at the Labuk river in North Sabah. See Fig. 1 for a map of the regions and wasp records.

**Region, *Macaranga* species**	Trees inspected	Trees colonised by wasps	Location of selected colonised trees
**Eastern Sabah**			
M. sect. *Pachystemon* sp.	1	0	
M. pearsonii	2	0	
**Main study area**			
M. cf. *indistincta*	9	2	
M. hypoleuca	84	8	
M. pearsonii	67	3	
M. winkleri	16	0	
**North Sabah**			
M. sect. *Pachystemon sp.*	11	0	
M. hypoleuca	6	0	
M. indistincta	3	0	
M. lamellata	4	0	
M. motleyana	6	0	
M. pearsonii	3	1	5°40'50"N, 117°05'30"E
**Northwest Sabah**			
M. cf. *glandibracteolata*	1	1	6°02'30"N 116°12'50"E
