# Supplementary material for: Exploitation of an ant-plant mutualism by a cavity-nesting wasp
Source: PeerJ. 2026 Apr 15;14:e20984. doi: 10.7717/peerj.20984 (PMC13091584; doi:10.7717/peerj.20984)
Supplement: Supplemental Information 2 [file peerj-14-20984-s002.pdf]

Supplemental Table S2. Ant colony size (ordinal categorical variable) as predicted by plant main stem diameter, total stem length, and wasp abundance (ordinal logistic regression), at the between tree level. For each model on each row, presence of coefficients (or "+" for categorical predictors), indicate model structure. Models representing all combinations of main effects are presented, ranked by *AICc*. * Indicates models that despite having Δ*AICc* < 2 relative to the best model, included only a single additional predictor variable, and hence were not considered during interpretation of results.

**model no.**	**model formula**	**diameter (*D*)**	**length (*L*)**	**wasp abundance (*W*)**	***df***	**log*L***	***AICc***	**Δ*AICc***	**weight**
6	*A ~ D + W*	2.719		-0.10080	6	-117.955	248.6	0	0.448
8*	*A ~ D + L + W*	3.465	-0.003172	-0.08998	7	-116.974	248.9	0.28	0.389
4	*A ~ D + L*	3.156	-0.003866		6	-119.453	251.6	3	0.1
2	*A ~ D*	2.163			5	-121.016	252.5	3.91	0.063
3	*A ~ L*		0.004579		5	-130.25	271	22.38	0
7	*A ~ L + W*		0.005418	-0.05272	6	-129.466	271.6	23.02	0
1	*A ~ 1*				4	-137.485	283.3	34.68	0
5	*A ~ W*			0.01967	5	-137.342	285.2	36.56	0

Supplemental Table S3. Ant colony size (ordinal categorical variable) as predicted by section length, stem type (main stem vs. side branch (binary)), and wasp abundance (cumulative link mixed model), at the within tree level.
For each model on each row, presence of coefficients (or "+" for categorical predictors), indicate model structure. Models representing all combinations of main effects are presented, ranked by *AICc*. *Indicates models that despite having Δ*AICc* < 2 relative to the best model, included only a single additional predictor variable, and hence were not considered during interpretation of results.

**model no.**	**model formula**	**length (*L*)**	**stem type (*T*)**	**wasp abundance (*W*)**	***df***	**log*L***	***AICc***	**Δ*AICc***	**weight**
2	*A ~ L*	0.01653			6	-153.426	319.4	0.00	0.393
4*	*A ~ L + T*	0.02101	+		7	-152.473	319.7	0.29	0.341
6	*A ~ L + W*	0.01597		0.02276	7	-153.353	321.5	2.05	0.141
8	*A ~ L + T + W*	0.02047	+	0.02669	8	-152.373	321.7	2.31	0.124
7	*A ~ T + W*		+	0.09683	7	-160.553	335.9	16.44	0
5	*A ~ W*			0.1219	6	-161.695	335.9	16.54	0
3	*A ~ T*		+		6	-162.022	336.6	17.19	0
1	*A ~ 1*				5	-164.221	338.8	19.43	0

Supplemental Table S4. Leaf area loss (as a proportion in the range 0-1) as predicted by plant diameter, ant colony size (ordinal categorical variable), section length, and wasp abundance (Beta regression) at the between tree level.

Models representing all combinations of main effects are presented, ranked by *AICc*. *Indicates models that despite having Δ*AICc* < 2 relative to the best model, included only a single additional predictor variable, and hence were not considered during interpretation of results.

model no.	Int.	stem diameter	ants	total stem length	wasp abundance	*df*	log*L*	*AICc*	Δ*AICc*	weight
1	-1.202					2	58.248	-112.4	0.00	0.221
9*	-1.242				0.02196	3	58.97	-111.7	0.67	0.158
5*	-1.259			3.50×10^{-4}		3	58.412	-110.6	1.78	0.09
2*	-1.278	0.068940				3	58.339	-110.5	1.93	0.084
11	-1.314		+		0.03204	7	62.756	-110.4	1.94	0.084
3	-1.246		+			6	61.341	-109.9	2.50	0.063
13	-1.25			6.00×10^{-5}	0.02125	4	58.973	-109.6	2.81	0.054
10	-1.237	0.004866			0.02218	4	58.97	-109.6	2.82	0.054
4	-1.547	0.2082	+			7	61.938	-108.8	3.58	0.037
6	-1.247	0.018740		4.10×10^{-4}		4	58.414	-108.5	3.93	0.031
7	-1.368		+	5.50×10^{-4}		7	61.703	-108.3	4.05	0.029
12	-1.437	0.09087	+		0.02805	8	62.852	-108.3	4.07	0.029
15	-1.343		+	1.50×10^{-4}	0.03030	8	62.779	-108.2	4.22	0.027
14	-1.224	0.041200		1.80×10^{-4}	0.02152	5	58.984	-107.4	4.98	0.018
8	-1.552	0.2175	+	4.00×10^{-5}		8	61.938	-106.5	5.90	0.012
16	-1.46	0.1374	+	2.00×10^{-4}	0.02842	9	62.87	-106.0	6.40	0.009
