# Supplementary figures and images for: Exploitation of an ant-plant mutualism by a cavity-nesting wasp
Source: PeerJ. 2026 Apr 15;14:e20984. doi: 10.7717/peerj.20984 (PMC13091584; doi:10.7717/peerj.20984)

(a)

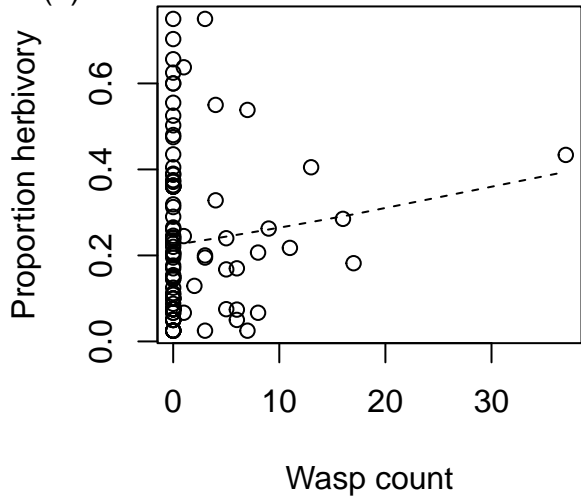

(b)

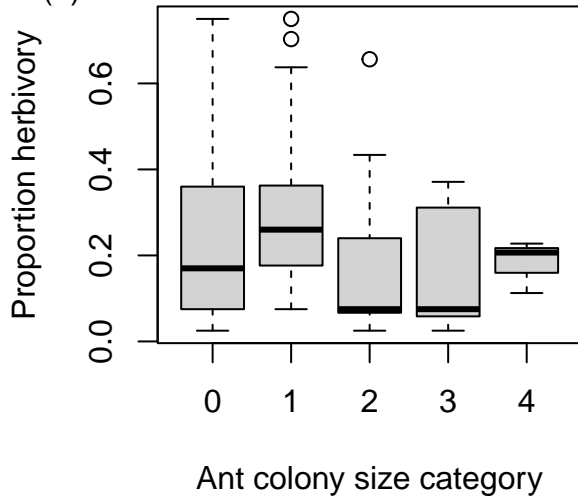

Supplement: Supplemental Information 3 — Proportion of leaf area on M. pearsonii plants with herbivory damage as predicted by (a) number of wasps and (b) ant colony size category. Sample sizes for ant colony size categories: 0: n=69; 1: n=17; 2: n=13, 3: n=11, 4: n=3. Note that a model with no predictors and only an intercept had equally good support. [file peerj-14-20984-s003.pdf]

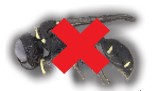

Supplement: Supplemental Information 4 [file peerj-14-20984-s004.zip › Lestina et al (code and data)/No wasp.jpg]

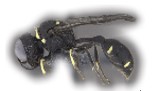

Supplement: Supplemental Information 4 [file peerj-14-20984-s004.zip › Lestina et al (code and data)/Wasp.jpg]
